# Rooks perceive support relations similar to six-month-old babies

**DOI:** 10.1098/rspb.2009.1456

**Published:** 2009-10-07

**Authors:** Christopher D. Bird, Nathan J. Emery

**Affiliations:** 1 Subdepartment of Animal Behaviour, Department of Zoology, University of Cambridge, Cambridge, UK; 2 School of Biological and Chemical Sciences, Queen Mary University of London, London, UK

**Keywords:** rooks, cognition, support, expectancy violation

## Abstract

Some corvids have demonstrated cognitive abilities that rival or exceed those of the great apes; for example, tool use in New Caledonian crows, and social cognition, episodic-like memory and future planning in Western scrub-jays. Rooks appear to be able to solve novel tasks through causal reasoning rather than simple trial-and-error learning. Animals with certain expectations about how objects interact would be able to narrow the field of candidate causes substantially, because some causes are simply ‘impossible’. Here we present evidence that rooks hold such expectations and appear to possess perceptual understanding of support relations similar to that demonstrated by human babies, which is more comprehensive than that of chimpanzees.

## 1. Introduction

The physical world is governed by unobservable forces such as gravity. These forces govern how objects interact and impose causal regularities. There is debate about whether any non-human animals appreciate unobservable forces (Povinelli 2000; Emery & Clayton 2009); however, there is evidence to suggest that some animals, including some species of corvid such as rooks and New Caledonian crows, are able to appreciate causal regularities when solving technical problems (Seed *et al*. 2006; Taylor *et al*. 2009).

The causal structure of the world may be represented by formulating expectations about what is possible and what is not, and so may be used to build more general rules about physical concepts, such as support. Certain conditions are required for support, such as contact between an object and its supporting platform. Only certain types of contact are appropriate for support and there must be a sufficient amount of contact between the surfaces. Solving support-related problems may involve an understanding of means–end relations or may be based on the specific perceptual features related to the spatial arrangement of object and support (Povinelli 2000). The causal structure of the world cannot be perceived directly (one cannot ‘see’ gravity) and must be inferred from the spatio-temporal relationships between objects (Chappell 2006).

The development of support concepts in human infants has been well studied using the ‘expectancy violation paradigm’, first introduced by Spelke (1985). When an unlikely or impossible event occurs, the observer's expectations are violated and they are surprised, looking for longer at the event. The increase in looking time may represent time taken to search for the cause of the unexpected event, or the time taken to update old expectations and incorporate new information. Although the interpretation of perceptual and cognitive capacities revealed by the expectancy violation paradigm has been debated (Thelen & Smith 1994; Bogartz *et al*. 1997, 2000; Haith 1998; Rivera *et al*. 1999; Cashon & Cohen 2000; Munakata 2000; Schilling 2000), many researchers have successfully employed the technique to investigate physical knowledge in humans and non-human primates (e.g. Hauser & Carey 1998; Munakata *et al*. 2000; Santos & Hauser 2002; Wang *et al*. 2004).

Baillargeon and colleagues found that an understanding of support relations follows a sequential developmental progression in humans: infants of just three months of age realize that objects cannot remain stable without contact; however, not until they are 4.5 months old do they realize that an appropriate type of contact is required, and only past the age of 6.5 months are they aware that the amount of contact must also be considered (Baillargeon *et al*. 1992, 1995; Needham & Baillargeon 1993). Infants of these ages showed this reasoning only with dynamic support violations, with static presentations failing to induce a response in infants under six months old (Baillargeon 1994). This dynamic test has also been used with adult chimpanzees, who understood the need for contact and the amount of contact, but not that the type of contact must also be considered (Cacchione & Krist 2004).

Expectancy violation has not been used as a paradigm with birds, partly owing to the difficulty in identifying where a bird is looking (Martin 2007), and their capability to switch from lateral to frontal vision depending on their distance from the observed object (Dawkins 2002). We therefore used the natural tendency of rooks to look through small holes (Bird & Emery 2008) to record the frequency and duration of their looking behaviour when viewing static images of possible and impossible support relations.

## 2. Material and methods

### (a) Subjects and housing

Subjects were seven adult rooks (*Corvus frugilegus*), 4 years of age at the time of testing (one male and six females). These were part of a hand-raised group colony of 12 rooks, kept in large outdoor aviaries (20 × 8 × 3 m) at the University of Cambridge's Subdepartment of Animal Behaviour at Madingley, UK. All subjects had previous experience with image presentation on an LCD screen (Bird & Emery 2008) and all took part in four consecutive experiments.

### (b) Experimental set-up

Subjects were tested individually in a naturally lit single chamber (3 × 1.6 × 1.5 m) in which they were visually and physically isolated from the rest of the group (see the electronic supplementary material, fig. S1). A box was mounted on the front wall of the chamber in which a hole (2 cm diameter) was cut. At the back of the box, at a distance of 50 cm from the holes, the subjects could see a 24-inch LCD monitor screen (LCD SM244 T, Samsung Electronics, South Korea). Birds could access this hole by sitting in the middle of a perch 1.65 m high, such that the hole was at their natural eye level.

### (c) Stimuli

**Figure 1.**
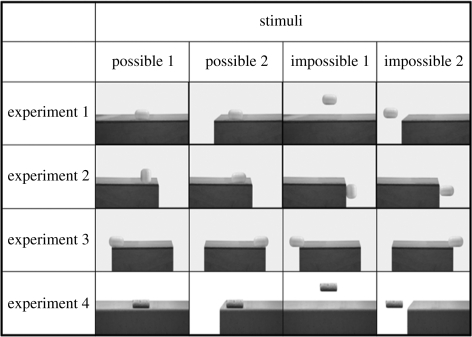
Picture stimuli used for experiments 1–4. Experiment 1: contact or no contact. Experiment 2: type of contact. Experiment 3: amount of contact. Experiment 4: contact or no contact (novel objects). When presented to the subjects, stimuli were presented in colour.

Each experiment presented four picture stimuli, each consisting of an object and supporting platform in varied spatial arrangements (figure 1). Two stimuli displayed the object in a possible support position, while the other two stimuli displayed the object in an impossible arrangement where the object was not appropriately supported but remained suspended in this position. The familiar objects used in experiments 1–3 were a cylindrical plastic container (from the centre of a Kinder chocolate egg) and a wooden supporting platform. The birds had extensive experience of manipulating these cylinders on a wooden platform in the past. In contrast, the objects used in experiment 4 were entirely novel. These were a bottle cork and a metal platform, neither of which had previously been seen by these birds.

Stimuli were created from photographs of the objects and the supporting platforms taken at 6 MP resolution (SLR EOS 400D, Canon, Inc., Tokyo, Japan). The positional arrangements of the objects were varied using Adobe Photoshop CS2, and saved as JPG files for presentation. The stimuli were presented on the monitor using Microsoft Office PowerPoint 2007, controlled by a PC laptop (Vaio VGN-FZ11L, Sony Corp, Tokyo, Japan).

### (d) Procedure

Each experiment followed the same procedure but differed in the stimuli presented. Experiment 1 examined whether rooks are sensitive to the basic contact relation between an object and a support platform, namely that contact is required for adequate support. Experiment 2 examined whether rooks use the type of contact to distinguish between adequate and inadequate support relations—that is, the supporting platform contacts the object either from below (supporting) or to the side (non-supporting). Experiment 3 examined whether rooks are sensitive to the amount of contact that is required for sufficient support—that is, the supporting platform contacts either more than two-thirds of the object base (supporting) or less than one-third of the object base (non-supporting). The fourth experiment was designed to investigate whether support sensitivity was confined to familiarity or whether similar results would be found with novel objects.

During trials, each stimulus was presented consecutively in a pseudo-randomized order, remaining on-screen for 60 s (following the bird's first look), separated by 30 s of black screen. Subjects received four trials per experiment, such that each stimulus appeared in one of the four positions only once, so avoiding pseudo-replication and controlling for any effects of stimulus position.

### (e) Recording and coding

All trials were video recorded remotely via a camera (Atom Dome, Model AHC, CSP Technology Ltd, Scunthorpe, UK) and later scored frame-by-frame (using Observer software v. 5.0, Noldus Information Technology, Wageningen, The Netherlands) without information on which stimulus was being viewed by which bird. We recorded how often the bird looked through the hole at the stimuli, and for how long. The bird's first look was noted in real time, but the duration of this look and all subsequent looks were coded from video.

### (f) Data analyses

Data were normalized using a log transformation correcting a positive skew. Data were then analysed in Genstat v. 10, using a generalized linear mixed model (GLMM) assessing the effect of stimulus type (possible/impossible) and trial number (and the interaction between the two) on looking behaviour. The experimental design meant that there were two levels of block structure: subject and sub-bird (a subdivision of subject indicating trials nested within subject).

## 3. Results

For each experiment, data were analysed treating the four stimuli independently. As there were no significant differences between the looking responses towards the two possible stimuli nor towards the two impossible stimuli for any of the measures (see the electronic supplementary material, table S1), we combined the data and examined whether there were differences between the possible and impossible categories. There was an effect of trial number on the mean look duration in experiment 1 (GLMM: *F*_3,18_ = 3.49, *p* = 0.037) but no effect of trial on any of the other measures or any of the other experiments. This reflects a strong initial interest in the stimuli on the first trial of the first experiment with some habituation afterwards. There was also an interaction between stimulus and trial on the first look and mean look duration of experiment 1 (GLMM: *F*_3,80_ = 3.21, *p* = 0.027; *F*_3,80_ = 3.58, *p* = 0.017) and on the first look of experiment 4 (GLMM: *F*_3,80_ = 4.78, *p* = 0.004), indicating that habituation depended on the stimulus type.

**Figure 2.**
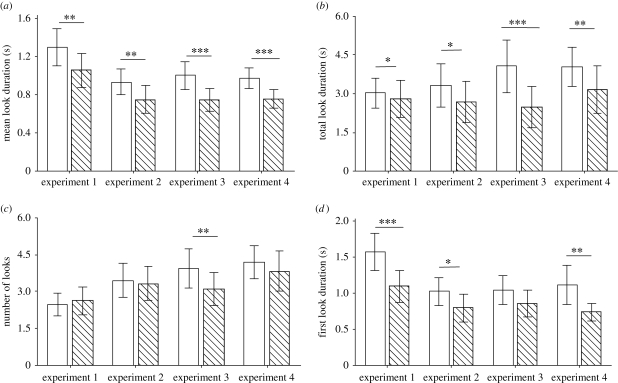
Looking responses (mean ± s.e.m.). (*a*) Mean look duration, (*b*) total look duration (per stimuli presentation), (*c*) number of looks and (*d*) first look duration. White bars indicate impossible stimuli, hashed bars indicate possible stimuli. *p < 0.05, **p < 0.01, ***p < 0.001.

All four experiments demonstrated a significant effect of stimulus category on looking response, with a greater looking response towards the impossible stimuli over the possible ones. This was represented in all four experiments by a greater mean look duration towards the impossible stimuli (GLMM: experiment 1, *F*_1,80_ = 8.13, *p* = 0.006; experiment 2, *F*_1,80_ = 8.08, *p* = 0.006; experiment 3, *F*_1,80_ = 13.64, *p* < 0.001; experiment 4, *F*_1,80_ = 13.75, *p* < 0.001; figure 2*a*) and by a greater total looking time towards the impossible stimuli (GLMM: experiment 1, *F*_1,80_ = 4.12, *p* = 0.046; experiment 2, *F*_1,80_ = 5.73, *p* = 0.019; experiment 3, *F*_1,80_ = 20.94, *p* < 0.001; experiment 4, *F*_1,80_ = 10.87, *p* = 0.001; figure 2*b*). All experiments except experiment 3 (amount of contact required) showed this difference from the very first look (GLMM: experiment 1, *F*_1,80_ = 13.78, *p* < 0.001; experiment 2, *F*_1,80_ = 6.44, *p* = 0.013; experiment 3, *F*_1,80_ = 2.49, *p* = 0.118; experiment 4, *F*_1,80_ = 7.08, *p* = 0.009; figure 2*d*); however, only in experiment 3 was there a difference in the frequency of looks, with more looks made towards the impossible than possible stimuli (GLMM: experiment 1, *F*_1,80_ = 0.00, *p* = 0.970; experiment 2, *F*_1,80_ = 0.45, *p* = 0.502; experiment 3, *F*_1,80_ = 11.42, *p* = 0.001; experiment 4, *F*_1,80_ = 2.59, *p* = 0.111; figure 2*c*). This may reflect the subtle differences between the possible and impossible stimuli in experiment 3.

## 4. Discussion

The results of experiments 1–3 suggest that rooks not only appear to understand the basic rule that contact is required for support, but also that appropriate support requires the correct type of contact and a sufficient amount of contact. Comparatively then, rooks appear to have a more comprehensive understanding of support than chimpanzees—taking into account the type of contact required, whereas chimpanzees did not—and to have an understanding of support equivalent to at least six-month-old infants. Further experimentation will help to reveal whether rooks, like infants of 13 months of age (Baillargeon 1995), are able to take into account the symmetry of the object when deciding the appropriate amount of contact for adequate support.

The results of experiment 4, whereby rooks responded to the impossibility of the no-contact stimuli even when the supported and supporting objects were entirely novel, suggest that the preferential looking responses are not due to a preference for perceptual novelty, but are rather based upon a set of general rules. Although the rooks had frequently seen the plastic cylinder supported on a wooden platform and therefore may have habituated to the possible picture, this could not explain the equivalent response to the novel objects. However, it is possible that the preference may be due to positional novelty independent of object familiarity. It may also seem probable that the decision as to whether something is impossibly supported may depend on additional rules such as whether the object is moving or is inanimate. Birds, for example may be seen defying gravity without contact to a supporting surface, yet this is not impossible. After seeing a novel self-propelled box move back and forth on an apparatus floor, 6.5-month-old human infants were found to be unsurprised if the box later remained stable when released either in mid-air or with an inadequate amount of contact with a platform (Luo *et al*. 2009).

The perceptual understanding of support has often been found at an earlier age in human infants than the age at which they can act on this understanding (Spelke *et al*. 1992; Hood *et al*. 2000). This may reflect a difference in the developmental timing of perceptual and motor skills. When the tasks are kept very simple, infants show the same responses in expectancy violation and action tasks (Hespos & Baillargeon 2008). ‘Perception–action’ dissociations have been found in non-human primates, persisting into adulthood (Hauser 2003). For example, adult rhesus monkeys consistently made search errors when having to take into account physical properties such as solidity and support in order to decide where to look for a food item (above or below a solid surface) that had been released above the surface and fallen out of sight (Hauser 2001). When shown the same event in an expectancy violation paradigm, rhesus monkeys looked for longer at the event when the food appeared below the surface than above, suggesting that when the food had apparently passed through the solid surface their expectations had been violated (Santos & Hauser 2002).

Rooks, however, have been found to take into account causal relations in their actions. Seed *et al*. (2006) found that seven out of eight rooks solved the two-trap tube task, a problem involving support, contact and gravity relations (see Visalberghi & Limongelli 1994, for original trap tube design). All seven rooks transferred their solution across a change in stimuli and one female solved further transfers of the test that could only be solved through abstracting a causal rule, as they shared no visual constant. Likewise, New Caledonian crows were found to solve the two-trap tube task using causal reasoning, avoiding the hole of the trap that the food would otherwise fall through and transferring this understanding to a trap-table that was visually different but was governed by the same underlying causal rules (Taylor *et al*. 2009). Rooks, like New Caledonian crows, are capable of using tools, and spontaneously solve new problems using tools based on an understanding of the properties of the tools and task affordances (Hunt 1996; Weir *et al.* 2002; Bird & Emery 2009*a*,*b*). The evidence of a comprehensive perceptual understanding of support in rooks further supports the claim that rooks are able to solve complex problems using advanced physical cognition rather than trial-and-error learning.
